# Evodiamine and Rutaecarpine as Potential Anticancer Compounds: A Combined Computational Study

**DOI:** 10.3390/ijms231911513

**Published:** 2022-09-29

**Authors:** Jingli Liu, Hui Guo, Jing Zhou, Yuwei Wang, Hao Yan, Ruyi Jin, Yuping Tang

**Affiliations:** Key Laboratory of Traditional Chinese Medicine Basic and New Drug Research of Shaanxi Province, School of Pharmacy, Shaanxi University of Chinese Medicine, Xianyang 712046, China

**Keywords:** evodiamine, rutaecarpine, anticancer, DFT calculations, molecular docking, molecular dynamic simulations

## Abstract

Evodiamine (EVO) and rutaecarpine (RUT) are the main active compounds of the traditional Chinese medicinal herb *Evodia* *rutaecarpa*. Here, we fully optimized the molecular geometries of EVO and RUT at the B3LYP/6-311++G (d, p) level of density functional theory. The natural population analysis (NPA) charges, frontier molecular orbitals, molecular electrostatic potentials, and the chemical reactivity descriptors for EVO and RUT were also investigated. Furthermore, molecular docking, molecular dynamics simulations, and the analysis of the binding free energies of EVO and RUT were carried out against the anticancer target topoisomerase 1 (TOP1) to clarify their anticancer mechanisms. The docking results indicated that they could inhibit TOP1 by intercalating into the cleaved DNA-binding site to form a TOP1–DNA–ligand ternary complex, suggesting that they may be potential TOP1 inhibitors. Molecular dynamics (MD) simulations evaluated the binding stability of the TOP1–DNA–ligand ternary complex. The calculation of binding free energy showed that the binding ability of EVO with TOP1 was stronger than that of RUT. These results elucidated the structure–activity relationship and the antitumor mechanism of EVO and RUT at the molecular level. It is suggested that EVO and RUT may be potential compounds for the development of new anticancer drugs.

## 1. Introduction

Cancer is one of the most serious diseases threatening the health and lives of people all over the world [1]. There is a strong need to develop more effective antitumor drugs and cancer therapy methods. Although there are more than 100 antitumor drugs in clinical application, most of them are ineffective at preventing cancer metastasis [2]. Therefore, developing new and more active anticancer compounds remains a challenge for medicinal chemistry [3]. The discovery and development of antitumor drugs from natural products, especially from plants, is currently a research hotspot [4,5,6]. For instance, camptothecin (CPT), isolated from the Chinese tree *Camptotheca acuminnata*, is a specific inhibitor of DNA topoisomerase 1 (DNA-TOP1), which is an important anticancer target [7,8,9,10].

DNA–TOP1 is a key enzyme that is indispensable for cellular DNA transcription and replication. It can maintain DNA topology by relaxing DNA supercoiling and catalyzing single-strand breakage and relinking during DNA replication [11]. The enzyme is composed of four domains: an N-terminal domain, core domain, linker region, and carboxyl-terminal domain. The core domain includes amino acids 215–635. The carboxy-terminal domain is composed of amino acids 713–765 and contains the active-site Tyr723. The core domain is tightly packed with the carboxyl-terminal domain to form the main structure of the enzyme [12]. Based on the structural analysis of the crystals structure, Stewart et al. proposed that the active site residues are Arg488, Arg590, His632, and Tyr723 [13].

Topoisomerase 1 (TOP1) inhibitors constitute an important class of anticancer drugs. TOP1 inhibitors can be divided into two types: repressors and poisons. Repressors can kill cells by inhibiting the binding of TOP1 to DNA cleavage sites. Poisons can capture DNA–TOP1 cleavable complexes and form a “road blocker” that prevents replication and leads to cell death [14]. Camptothecin (CPT) and its analogs are the classic TOP1-specific inhibitors, which are widely used as TOP1 poisons in clinical practice. However, there are still some limitations in the clinical application of CPT [15]. Therefore, the discovery of novel TOP1 inhibitors with improved pharmacological properties from natural products is crucial for the discovery and development of anticancer drugs.

**Figure 1:**
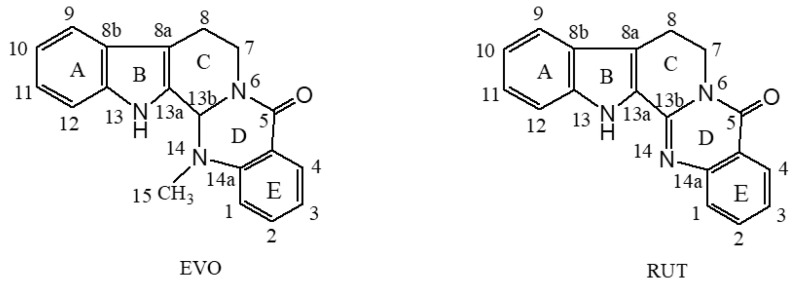
The molecular structures of evodiamine (EVO) and rutaecarpine (RUT). Ring labeling is represented by A, B, C, D, and E, while atom numbering is represented by numbers and lowercase letters.

Evodiamine (EVO) and rutaecarpine (RUT) are the main active components isolated from the Chinese herb *Evodia rutaecarpa* [16]. They are often used as chemical markers for the quality control of *E. rutaecarpa* [17]. These two components have been shown to possess various pharmacological effects, including anti-inflammatory, vasodilatory, anti-obesity, and antitumor effects [18,19,20]. The molecular structures of EVO and RUT are shown in Figure 1. They belong to the class of indolopyridoquinazolinone heterocycles consisting of five rings linked together. The A, C, D, and E rings are six-membered, and the B ring is five-membered. They have very similar geometries except for a methyl attached to the N14 atom of EVO. The crystal structures of EVO and RUT were determined using X-ray crystallography by Hirayama et al. [21], who reported that EVO takes a non-planar conformation, whereas RUT takes a planar conformation.

In recent years, EVO and RUT have aroused intense research interest owing to their potential anticancer activities [22,23]. Previously, our group synthesized EVO and RUT and studied their antitumor activities in vivo [24,25]. In order to study the inhibitory effects of EVO and RUT on different tumor cell lines, we used the MTT assay to evaluate the antitumor activities in vitro. Our results showed that the antitumor activity of EVO was greater than that of RUT [26].

Studies on the molecular mechanism indicated that the antitumor effects of EVO and RUT induce apoptosis and inhibit invasion and metastasis [27,28,29]. The inhibitory activity of EVO against TOP1 was previously investigated, and the results showed that EVO can inhibit TOP1 by stabilizing the enzyme–DNA covalent complex [30]. EVO was also identified as a TOP1 inhibitor by structure-based virtual screening [31]. Subsequently, evodiamine derivatives were synthesized as TOP1 inhibitors by introducing various groups at N-13. The results showed that N-benzoyl analogs exhibited stronger antiproliferative effects than evodiamine. Later, a library of evodiamine A ring, E ring, A, E ring disubstituted, and D ring derivatives were synthesized by Dong et al. [32]. The structure–activity relationship studies indicated that the introduction of one or two hydrophilic groups (OH and NH_2_) at the C3 and C10 positions could enhance the antitumor activity.

The TOP1 inhibitory activities of RUT and its derivatives have also been studied [33]. These inhibitory activities seemed to be stemming from the introduction of substitutions on the rings of RUT [34]. For example, 10-bromorutaecarpine and 3-chlororutaecarpine showed strong inhibitory effects on TOP1. However, the inhibitory mechanism of RUT against TOP1 is still unclear. Recently, the synthesis and anticancer activities of novel EVO and RUT semisynthetic derivatives and their molecular hybrids were reported [35]. The results showed that structural modification at position 13 of EVO and RUT did not improve antitumor activity.

Despite the promising antitumor activities of both EVO and RUT, the relationships between their respective molecular structures and antitumor activities, as well as their mechanisms of action, are not well understood at the molecular level. Computational chemistry methods can provide significant information at a low cost, which is superior to time-consuming and expensive experimental or clinical studies. DFT calculations can be performed to elucidate the relationship between the structure and biological activity of drugs, thereby providing a theoretical basis for rational drug design [36]. Molecular docking can be used to not only study the detailed interaction between the ligand (drug molecule) and its receptor (known target protein or active site) as well as predict its binding mode and affinity, but it can also be used for drug design [37]. Molecular dynamics (MD) can be used to help study the dynamic behavior of drug–protein interactions at the molecular level [38].

In the present study, density functional theory (DFT) was used to investigate the structure–antitumor relationship of EVO and RUT. Molecular docking was carried out to predict the binding mode between the ligand and the target, and molecular dynamics simulation was performed to evaluate the binding stability of the complex. Detailed computational studies of EVO and RUT will not only provide a clear understanding of the relationship between their molecular structure and anticancer activity but also their inhibitory mechanism against TOP1. It is therefore critical to gain theoretical insight into the antitumor activity and mechanism of EVO and RUT at the molecular level.

## 2. Results and Discussion

### 2.1. Antitumor Activities of EVO and RUT

The antitumor activities of EVO and RUT against the human cancer cell line Hepg2 were assessed using the 3-(4,5-dimethylthiazol-2-yl)-2,5-diphenyltetrazolium bromide (MTT) assay. The growth inhibition rate of tumor cells by drugs was obtained by calculating the percentage of dead cells. EVO and RUT showed dose-dependent inhibitory effects on the proliferation of Hepg2 cells (Figure S1). At a drug concentration of 100 μmol/L, the inhibition rate of EVO was 40.00 ± 6.16%, while the inhibition rate of RUT was only 10.70 ± 1.65%. This showed that the anti-tumor activity of RUT was stronger than that of RUT.

### 2.2. DFT Calculation Studies

There is an important relationship between the conformation of a drug molecule and its biological activity. When a drug interacts with a receptor, the geometry of the drug must be complementary to the conformation of the receptor [39]. Therefore, we performed calculations regarding the geometries of EVO and RUT using the DFT method to reveal the relationship between the structures and their activities. The fully optimized geometrical parameters of EVO and RUT in the gas and liquid phases are listed in Table S1. The optimized bond lengths and bond angles of the compounds in the gas phase and liquid phase were very similar. This suggests that solvation has little effect on the molecular geometries (Table S1).

In accordance with the X-ray structure, the EVO’s optimized geometry adopted a nonplanar conformation [21]. The optimized geometry of RUT was nearly planar. The difference between the structures of EVO and RUT lies in the methyl group attached to N14, which seems to be responsible for the nonplanar structure of EVO. Therefore, it may have contributed to the difference in antitumor activity between EVO and RUT.

#### 2.2.1. The Natural Population Analysis (NPA) Charge

The charge distribution on a drug molecule is closely related to its activity and interactions with other small molecules or biological macromolecules, especially with its targets. If the charge density distribution of the drug molecule precisely matches that of the specific receptor, the drug and receptor will be in close proximity, the interaction will increase, and the drug and receptor will readily form a complex to enhance its activity [40].

The NPA charges of EVO and RUT were determined by calculating the natural bond orbital (NBO) using the B3LYP/6-311++G (d, p) method; the charges are shown in Table 1. Electrophilic attacks were most likely to target negatively charged centers. However, positively charged centers were particularly susceptible to nucleophilic attacks. We observed that in EVO, C5 had the most positive charge and N6, O5, N13, N14, and C15 had the most negative charges. In RUT, we found that O5, N6, N13, and N14 had the most negative charges, while C5 was positively charged. This suggests that the C5 atom is the nucleophilic attack site for both EVO and RUT. This is due to the electronegativity of O5 and its strong electron-withdrawing ability, which gives C5 a positive charge. The lower positive charge of the C5 atom in EVO (0.334), when compared to RUT (0.676), indicates that the nucleophilicity of EVO is weaker. Moreover, the electrophilic sites were located on O and N atoms because O and N atoms have lone pair electrons. The greater negative charges of EVO’s O5, N6, N13, and N14 atoms than those of RUT indicate that EVO’s electrophilicity is stronger than that of RUT. Therefore, EVO can interact with the receptor as an electron donor, while RUT can interact as an electron acceptor.

#### 2.2.2. Molecular Electrostatic Potential (MEP)

**Figure 2:**
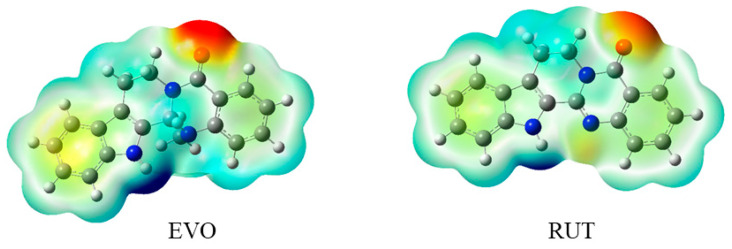
Molecular electrostatic potential map of evodiamine (EVO) and rutaecarpine (RUT). The molecular electrostatic potential is mapped on the iso-surface of electron density with an iso-value of 0.001. The MEP color ranges from −0.06 (red) to 0.06 (blue). The unit is au.

MEP is a helpful tool for predicting the chemical reactivity of a compound. The knowledge of a compound’s chemical reactivity can be used to describe the interaction of drugs with proteins [41]. The calculated MEP for EVO and RUT are plotted in Figure 2. Positive regions (colored in blue) are related to nucleophilic reactivity, whereas negative ones (colored in red) are related to electrophilic reactivity. The positive MEP was localized on the hydrogen atoms of the indole ring, indicating the site of nucleophilic reactivity. The negative MEP was distributed around the oxygen atom of the D ring, which may indicate the site of electrophilic reactivity. These negative MEP regions may facilitate H-bond formation or van der Waals interactions with receptors. Clearly, EVO has a higher negative electrostatic potential than RUT, which can explain why EVO has a stronger binding ability compared with that of RUT.

#### 2.2.3. Frontier Molecular Orbitals (FMOs)

FMOs are the molecular orbitals located in the outermost layer of electrons in molecules. They consist of the highest occupied molecular orbital (HOMO) and the lowest unoccupied molecular orbital (LUMO). They are the primary orbitals involved in the chemical reactivity of molecules. The FMOs can predict how the drugs interact with the proteins. A drug molecule interacts with its receptor by sharing orbital interactions during the binding process. The HOMO of a drug molecule can interact with the LUMO of active site residues. Similarly, the LUMO of a drug molecule can interact with the HOMO of a neighboring residue [42].

The higher the energy of the HOMO, the easier it is for the molecules to donate electrons. On the other hand, lowering the energy of the LUMO makes it easier for the molecules to accept electrons. The energy gap between HOMO and LUMO can influence the reactivity of molecules. The lower the energy gap, the less stable and more active the molecule, while the higher the energy gap, the more stable and less active the system [43]. The calculated energies of the HOMOs and LUMOs, as well as the energy gaps of EVO and RUT, are tabulated in Table S2. The results of the FMO energy analysis indicated that the energy gap of EVO (3.96 eV) was lower than that of RUT (4.47 eV). This may explain why the antitumor activity of EVO is greater than that of RUT.

**Figure 3:**
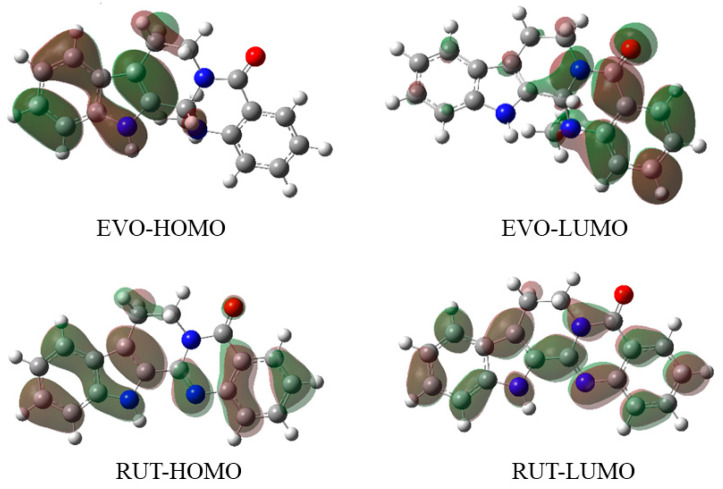
Calculated isodensity surface plots of the frontier molecular orbitals of evodiamine (EVO) and rutaecarpine (RUT). Red color represents positive orbitals and green color represents negative orbitals.

The iso-density surface plots of the FMOs for the studied molecules are depicted in Figure 3. Red represents positive orbitals and green represents negative orbitals. Evidently, the iso-density surface plots of EVO and RUT were dissimilar. For EVO, the HOMO was localized on the A and B rings, while the LUMO was localized on the D and E rings. For RUT, HOMO and LUMO were delocalized throughout the entire molecule. This indicates that RUT is a conjugated system, the planarity of which is determined by its molecular structure.

#### 2.2.4. Reactivity Descriptors

Reactivity descriptors that can describe chemical reactivity were derived from density functional theory calculations, including ionization potential (IP), electron affinity (EA), electronegativity (χ), chemical hardness (η), global softness (δ), and global electrophilicity index (ω) [44]. These parameters were calculated according to Koopman’s theorem, as shown in the following equations [45,46,47]:

$$IP=-E_{HOMO}$$

$$EA=-E_{LUMO}$$

$$\chi =-\frac{1}{2}\left(E_{HOMO}+E_{LUMO}\right)$$

$$\eta =-\frac{1}{2}\left(E_{HOMO}-E_{LUMO}\right)$$

$$\delta =\frac{1}{\eta }$$

$$\omega =\frac{\chi^{2}}{2\eta }$$

The value of electronegativity (χ) is a measure of the capacity of the molecule to attract electrons. The global hardness (*η*) of an atom is used to evaluate its resistance to charge transfer. The global softness (δ) of an atom or a group of atoms represents its ability to accept electrons. The electrophilicity index (*ω*) is a prediction of the stabilization energy of a molecule when its electrons are saturated, derived from electronegativity and chemical hardness [45]. The values of *χ*, *η*, *δ*, and *ω* for EVO and RUT at the B3LYP/6-311++G (d, p) level are tabulated in Table S2. These reactivity descriptors are useful for predicting drug–protein interactions. The values of *χ*, *δ*, and *ω* for EVO were higher than those of RUT, while η for EVO was lower than that of RUT. The harder a molecule is, the less reactive it is, and vice versa. Therefore, it was predicted that the interaction between EVO and the receptor was stronger than that of RUT.

### 2.3. Molecular Docking Studies

**Figure 4:**
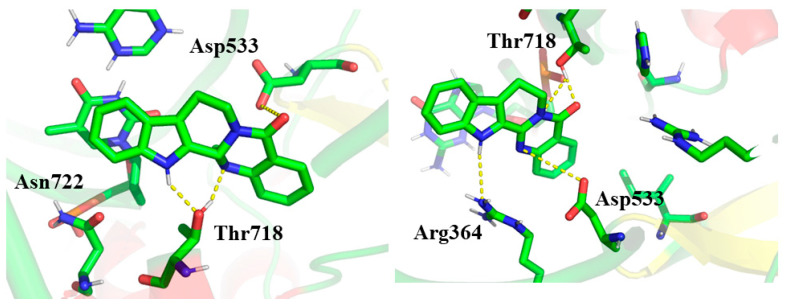
Binding mode of evodiamine (EVO) (left) and rutaecarpine (RUT) (right) with the TOP1–DNA complex. The ligand and relevant amino acids are represented by a stick diagram (green for carbon atom, red for oxygen atom and blue for nitrogen atom), while TOP1-DNA is depicted by a cartoon diagram. Hydrogen bond interactions are shown in yellow dotted lines.

Molecular docking is one of the most widely used techniques for examining structure–activity relationships and biological activity in drug discovery [48]. It helps to provide detailed information for studying the binding mode and interactions between a ligand and its receptor [49]. To better understand the antitumor mechanism of EVO and RUT at the molecular level, we employed the molecular docking technique to study their binding modes of inhibition against the protein TOP1. The binding patterns of EVO and RUT with TOP1 are shown in Figure 4. The dotted line represents the hydrogen bond interaction between ligands and receptors.

In addition to hydrogen bonding, p–p stacking interactions between DNA bases and aromatic rings of the ligand are crucial for DNA intercalation. Similar to the crystal structure of CPT, our docking results indicated that the ligand EVO intercalates at the cleaved DNA binding site parallel to the axis of base pairing [50]. The A and B rings of EVO formed base–stacking interactions with DNA base pairs, contrary to the binding mode predicted by Dong et al. [31]. At the same time, we found that EVO can form hydrogen bonds with the active site residues Thr718, Asn722, and Asp533. As a result, EVO, TOP1, and DNA formed a stable ternary complex via hydrogen bonding, van der Waals forces, and base stacking interactions. There was a hydrogen bond with a length of 2.12 Å between the hydrogen atom of the B ring and the hydroxyl group of Thr718. Another hydrogen bond with a length of 2.06 Å was formed between the nitrogen atom attached to a methyl of the D ring and the hydroxyl group of Thr718. Additionally, two hydrogen bonds existed between EVO and Asp533. One was positioned between the nitrogen atom of the C ring and the carboxyl group of Asp533, and the other was positioned between the oxygen atom of the D ring and the carboxyl group of Asp533.

Based on docking results, the ligand RUT also bound at the cleaved DNA binding site, similar to the intercalating pattern of EVO binding to TOP1. As a result, TOP1, DNA, and RUT formed a stable ternary complex. The A and B rings of RUT formed p–p stacking interactions with the DNA base pairs. Furthermore, the stability of the TOP1–DNA–RUT complex was enhanced by H-bond interactions with the active site residues. RUT formed H-bonds with the residues Arg364 and Thr718, which had previously been identified [51]. RUT formed one H-bond of length 2.90 Å with the residue Arg364, which was absent in the TOP1–DNA–EVO complex. In addition, two hydrogen bonds were formed between Thr718 and RUT. One was between the carbonyl oxygen atom of the D ring and the hydroxyl group of Thr718 with a length of 1.73 Å. The other one occurred between the nitrogen atom of the D ring and the hydroxyl group of Thr718 with a length of 2.77 Å.

Both EVO and RUT can intercalate at the binding site parallel to the axis of base pairing, which is similar to the known TOP1 inhibitor CPT. Furthermore, these two compounds are also stabilized by H-bonding interactions and p–p stacking interactions. Both EVO and RUT can form two stable hydrogen bonds with Thr718, indicating that it is an important interacting residue in the active site. The binding energy of EVO with TOP1 was −8.79 kcal·mol^−1^, which is lower than that of −8.32 kcal·mol^−1^ for RUT, thereby indicating that EVO might be a better inhibitor of TOP1 than RUT. This could be attributed to the stronger antitumor activity of EVO compared with RUT. The binding energy of the known inhibitor CPT against TOP1 was −10.81 kcal·mol^−1^. It was shown that the antitumor activities of EVO and RUT were slightly weaker than that of CPT.

### 2.4. MD Simulation Studies

**Figure 5:**
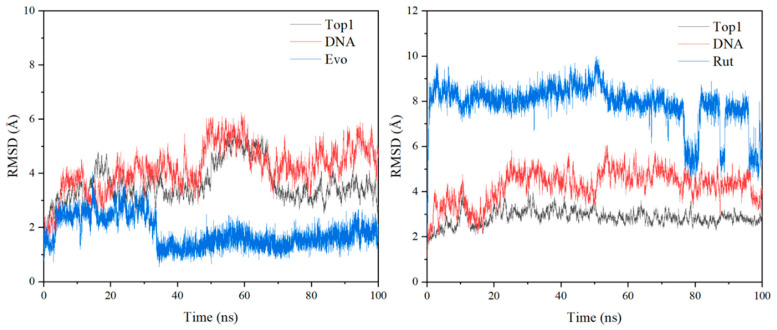
The root-mean-square deviation (RMSD) values of heavy atoms of protein, DNA, and inhibitor in 100 ns molecular docking simulations for the TOP1/EVO system (**left**) and TOP1/RUT system (**right**).

To evaluate the stability of TOP1 docked with EVO and RUT using AMBER 12, a total of 100 ns of MD simulations was performed. The root-mean-square deviation (RMSD) values of heavy atoms of protein, DNA, and inhibitor of the TOP1/EVO system and TOP1/RUT systems are illustrated in Figure 5. Our results indicated fewer fluctuations in protein, DNA, and inhibitor structure during MD simulation, indicating that the TOP1–DNA–ligand ternary complex was stabilized. The ligand remained securely at the cleaved DNA binding site. There is only one stable hydrogen bond between EVO N13 and Thr718 with 96% occupancy during the 100ns MD simulations. This suggests that when the structural modification is substituted at the N13 position, this hydrogen bond needs to be preserved or replaced by a new hydrogen bond.

To compare the conformational changes of the enzyme before and after ligand binding, we also performed MD simulations on TOP1–DNA of the unbound ligand. The analyzed RMSD values are shown in Figure S2. There was very little conformational change in the enzyme and DNA before and after the drug bound to the enzyme. This showed that the structure of the enzyme and DNA was stable throughout the MD simulations.

The root-mean-square fluctuation (RMSF) values of TOP1, TOP1/EVO, and TOP1/RUT are given in Figure S3. RMSF gives a characterization of the flexibility of various regions of the protein based on the fluctuation of all amino acids. The amino acid residues at positions 228–252, 261–276, 283–338, 512–514, 603−612, and 663–689 of TOP1 presented with large fluctuations (RMSF > 1.00). The linker region comprising amino acids 636–712 showed a certain degree of flexibility, which was in good agreement with the crystal structure [52]. The radius of gyration represents a degree of compaction, or extensibility of the ligand complexed within the binding pocket during the MD simulation study. The radius of gyration of protein, DNA, and ligand in 100 ns MD simulations for the TOP1/EVO system and TOP1/RUT system is illustrated in Figures S4 and S5, indicating that the systems have reached a stable state.

The surface of the protein, DNA, and ligand in 100 ns MD simulations for the TOP1/EVO system and TOP1/RUT system are shown in Figures S6 and S7. Throughout the entire MD simulation, the surface of the protein showed less fluctuation in the binding pocket. The surface of the ligand was very low, indicating that it was completely buried within the binding pocket. This binding model can explain that most of the solvent-accessible surface of the ligand is covered by DNA, which is consistent with the X-ray structural analysis [50]. We identified the conformations of the TOP1/EVO and TOP1/RUT systems. They were represented by the snapshots taken at 10, 20, 30, 40, 50, 60, 70, 80, 90, and 100 ns from the MD trajectory. They are depicted and aligned in Figures S8 and S9. It is evident that the protein conformation was stable when the ligand was bound at the active site. Our calculational binding models can explain several of the known structure–activity relationships of EVO and RUT. Previous structural modifications have shown that substitutions at the C10 and the N13 position of EVO and RUT, in some cases, can improve the in vivo activity of the drug [31,32,34]. For example, 10-hydroxyl evodiamine and 10-Br rutaecarpine showed good inhibitory activities against TOP1. The binding models suggested that these positions face the major groove of DNA, so modifications at these positions to improve solubility, stability, and bioavailability will not sterically affect drug binding. Examination of the ternary complex showed that there is sufficient space within the binding pocket to accommodate the substitution on the E ring. Our calculated structural model supported the improvement of the activity of the drug by introducing substituents on the E ring [32].

### 2.5. Binding Free Energies

The binding free energy can quantitatively predict the binding ability between the ligand and the receptor. The calculated binding free energies for the two complex systems are summarized in Tables S3 and S4. The binding free energy of EVO with TOP1 was −35.97 kcal·mol^−1^, which was lower than that of RUT, which was −33.34 kcal·mol^−1^. This suggests that EVO might be a better inhibitor of TOP1 than RUT, and this may account for EVO’s stronger antitumor-activity. This conclusion is consistent with the results predicted by previous DFT calculations.

The inhibitor binding is typically impacted by polar (Eele + EGB) and nonpolar (Evdw + Esurf) terms. The polar terms for EVO/TOP1 and RUT/TOP1 were 18.70 kcal/mol and 15.46 kcal/mol, respectively. Positive electrostatic contribution values indicate unfavorable polar interactions between the ligand and receptor for binding. Thus, for EVO/TOP1 and RUT/TOP1 systems, polar interactions are unfavorable for their binding.

For EVO/TOP1 and RUT/TOP1, the total nonpolar values were −54.67 kcal/mol and −48.79 kcal/mol, respectively. Therefore, hydrophobic interaction is the main factor responsible for the binding of EVO and RUT with TOP1. In addition, it can be demonstrated that EVO has a stronger binding ability than RUT. Thus, EVO exhibited stronger antitumor activity than RUT.

### 2.6. Free Energy Decomposition

The free energy calculation suggested that the nonpolar term played an important role in complex binding. The per-residue free energy decomposition strategy can be used to analyze ligand–receptor interactions. The MM/GBSA decomposition protocol was used to calculate the interaction energy between the ligand and each residue (Tables S5 and S6).

Due to hydrogen bonding with EVO, the residue T718 contributed < −3.00 kcal/mol for EVO/TOP1. Particularly, some hydrophobic residues had significant subtotal binding free energies. DT10 and DT11, which are located at the cleaved DNA binding site, were the most favorable contributions. The hydrophobic interaction formed between RUT and DNA was primarily due to the contributions of DT10, DT11, and DA35 to RUT/TOP1. The majority of residues that contributed significantly to overall binding were located at the cleaved DNA binding site.

## 3. Materials and Methods

### 3.1. Cell Culture and MTT Assay

Hepg2 cells in the logarithmic growth phase were collected and counted with a hemocytometer to maintain the cell density per well at 5 × 10^3^ cells/well. The plated 96-well plate was incubated at 37 °C with 5% CO_2_ for 24 h.

Hepg2 cells in the logarithmic growth phase were treated with different kinds and concentrations of drugs. To this end, 200 μL of drug-containing medium or blank medium was added to each well, and six duplicate wells were set for each drug group. After incubation at 5% concentration at 37 °C for 3.5 h, the absorbance of each well at a wavelength of 450 nm was read with a thermo microplate reader.

### 3.2. Density Functional Theory (DFT) Calculations

The molecular geometries of EVO and RUT in the gas phase were optimized at the B3LYP/6-311++G (d, p) level using the DFT method. At the same level of theory, frequency calculations were performed on the optimized geometries of EVO and RUT to characterize the nature of the stationary points. The frequency calculation results showed that there are no imaginary frequencies in the optimized structures of EVO and RUT, indicating that their structures are stable. The two compounds were also optimized in aqueous solution by using the polarizable continuum model (PCM) [53]. All calculations were carried out using the Gaussian 09 program package [54].

### 3.3. Molecular Docking

Molecular docking calculations were performed using the AUTODOCK 4.2 program [55]. The X-ray crystal structure of TOP1 in complex with the poison CPT and covalent complex with a 22 bp DNA duplex was chosen as the initial docking structure, which can be obtained from the Protein Data Bank (PDB:1T8I) [50]. The crystal structure has a resolution of 3.00 Å, 592 amino acid residues, and only a single chain (chain A). In addition, the structures of EVO and RUT are highly similar to those of CPT. To compare with the known inhibitor CPT, we also performed a docking study of CPT with TOP1. AutoDock Tools were used to examine and prepare the ligands and receptors files. The protein and DNA were considered to be rigid molecules, and the conformation of the ligand was set to flexible. A grid of 44, 44, and 44 points in the x, y, and z directions, with a grid spacing of 0.375 A˚, was built centered on the mass of the receptors. Docking was carried out using the Lamarckian genetic algorithm. The docked compounds were subjected to 50 runs of Autodock search, with 500,000 steps of energy minimization and all parameters being the same for each docking.

### 3.4. Molecular Dynamics (MD) Simulations

The initial structure of the MD simulation was derived from the optimal conformation for docking. A total of 100 ns MD simulations was performed. The parameter file for ligands was generated using the Antechamber module. The Amber ff14SB force field [56] was applied to TOP1 and DNA. The GAFF force field [57] was applied to ligand molecules. The system was solvated in a water box with an explicit TIP3P water model. The water box’s margin distance was set to 8 Å to ensure that the system was fully immersed in the solution. We determined the total charge of the system and added Na^+^ to neutralize it. Following energy minimization, the complex model was heated in the NVT ensemble from 0 K to 300 K for 250 ps. The NPT ensemble underwent equilibrium for 50 ps at 300 K and 1 atm of constant pressure. Finally, 5000 frames were extracted from the last 5 ns as the average conformation of MD equilibrium for analysis. All dynamics simulations were carried out in AMBER 12 [58] using the PMEMD CUDA version. The CPPTRAJ module [59,60] was used to analyze the MD trajectory data.

### 3.5. Binding Free Energy Calculation

The binding free energies between the two alkaloids and TOP1 were calculated using the molecular mechanics-generalized Born surface area (MM/GBSA) method [61,62]. The energy term for each complex system was calculated by taking a statistical average of the last 20 ns of the MD trajectory over 200 frames. To gain insights into the contribution of each residue, the total binding energy between TOP1 and the ligand was decomposed with the MM/GBSA binding free energy decomposition, without consideration of the contribution of entropies [63]. The energy calculation was performed using the MMPBA.py program [64].

## 4. Conclusions

We conducted computational studies on EVO and RUT as potential anticancer compounds. DFT, molecular docking, and molecular dynamic simulation studies were performed on EVO and RUT as TOP1 inhibitors. The DFT results showed that EVO had a lower energy gap E, higher electronegativity χ, and electrophilicity index ω than RUT. It could be concluded that these parameters determined and influenced the antitumor activity of these two compounds to some extent. Molecular docking showed that EVO and RUT could bind at the cleaved DNA binding site via H-bonding, van der Waals, and p–p stacking interactions, indicating that they may be potential TOP1 inhibitors. A total of 100 ns MD simulations was carried out to validate the binding stability of the TOP1–DNA–ligand complex. The simulations showed that the ligand remained secure at the cleaved DNA binding site. The binding free energies of EVO/TOP1 and RUT/TOP1 were −35.97 kcal·mol^−1^ and −33.34 kcal·mol^−1^, respectively. It was concluded that EVO had stronger binding ability than RUT with TOP1. Therefore, EVO outperformed RUT in antitumor activity. In conclusion, we elucidated the structure–activity relationship and antitumor mechanism of EVO and RUT at the molecular level.

## Figures and Tables

**Table 1:** Natural population analysis (NPA) charge on the atoms of evodiamine (EVO) and rutaecarpine (RUT).

Atom	EVO	RUT	Atom	EVO	RUT
C1	−0.302	−0.190	C8	−0.737	−0.407
C2	−0.182	−0.184	C9	−0.745	−0.183
C3	−0.268	−0.209	C10	−0.592	−0.223
C4	−0.616	−0.148	C11	−0.226	−0.194
C5	0.334	0.676	C12	−0.284	−0.233
N6	−0.675	−0.474	N13	−0.602	−0.524
C7	−0.299	−0.153	N14	−0.645	−0.525
O5	−0.713	−0.622	C15	−0.768	-

## Data Availability

The data sets supporting the results of this article are included within the article.

## Supplementary Materials

The following supporting information can be downloaded at: https://www.mdpi.com/article/10.3390/ijms231911513/s1.
