# Supplementary material for: Evodiamine and Rutaecarpine as Potential Anticancer Compounds: A Combined Computational Study
Source: Int J Mol Sci. 2022 Sep 29;23(19):11513. doi: 10.3390/ijms231911513 (PMC9570036; doi:10.3390/ijms231911513)
Supplement: Supplementary file 1 [file ijms-23-11513-s001.zip › ijms-1883499-supplementary.pdf]

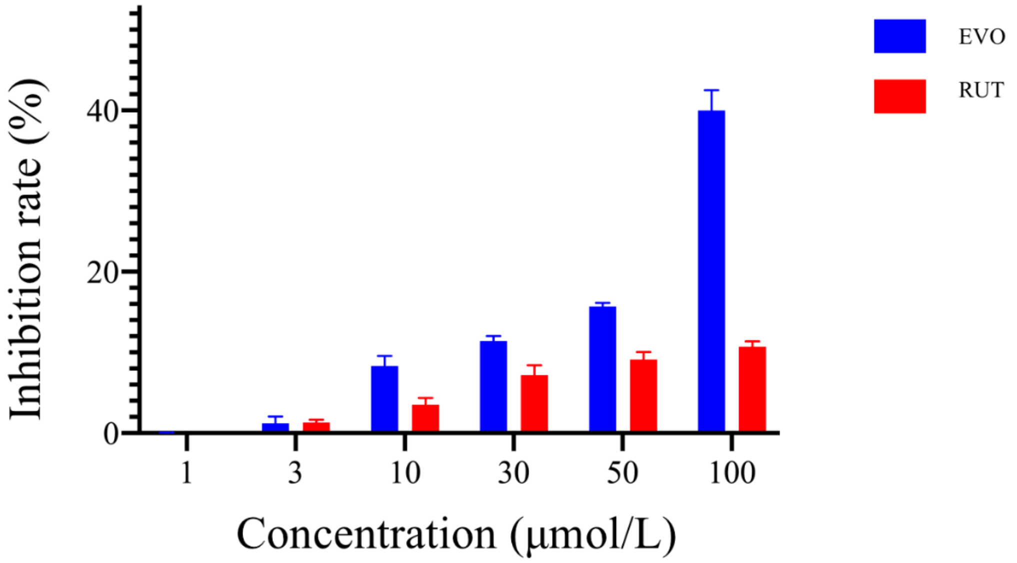

Figure S1. Antiproliferative effects of EVO and RUT on Hepg2 tumor cells at different drug concentrations.

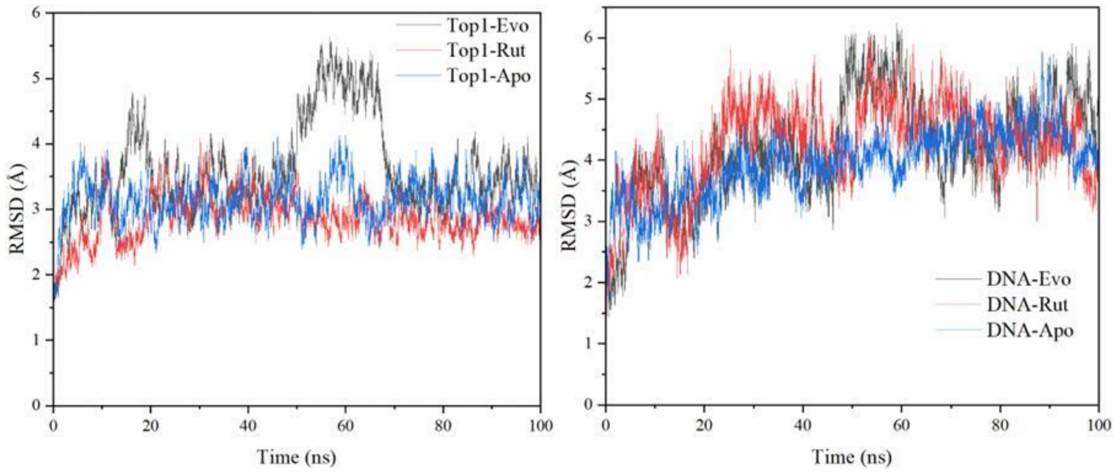

Figure S2. The RMSD of Top1-Apo, Top1-Evo, Top1-Rut, DNA-Apo, DNA-Evo, and DNA-Rut complexed systems.

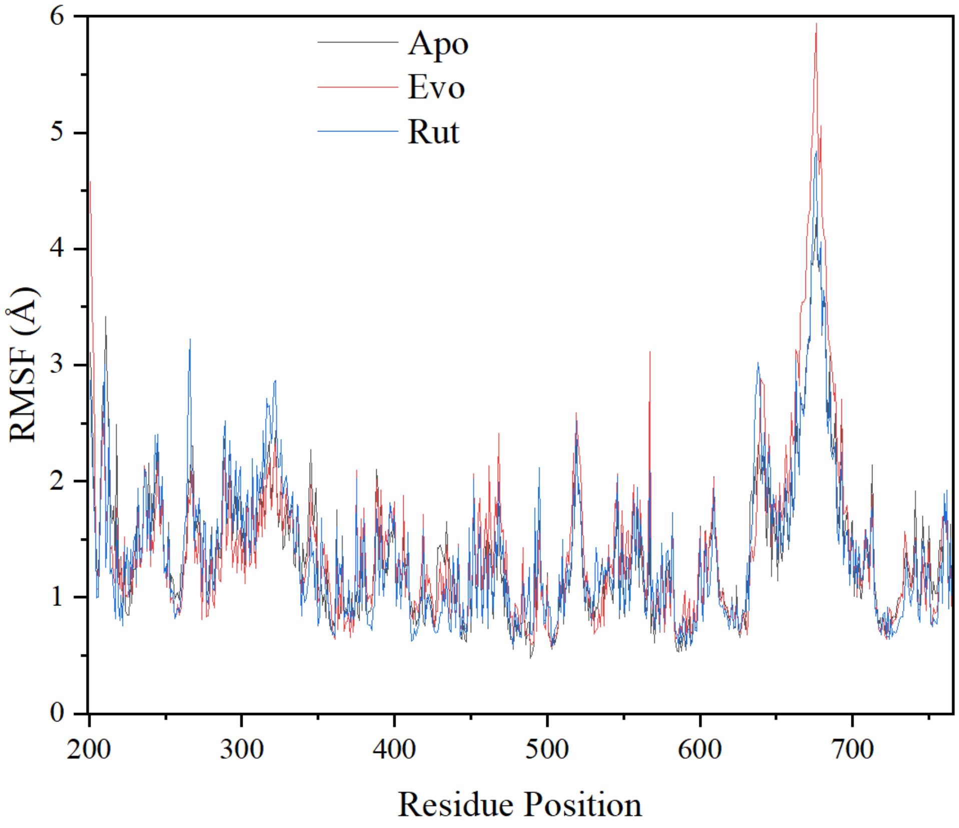

Figure S3. RMSF variations of the Cα atom of TOP1, Evo-TOP1, and Rut-TOP1 complexed systems from MD simulation.

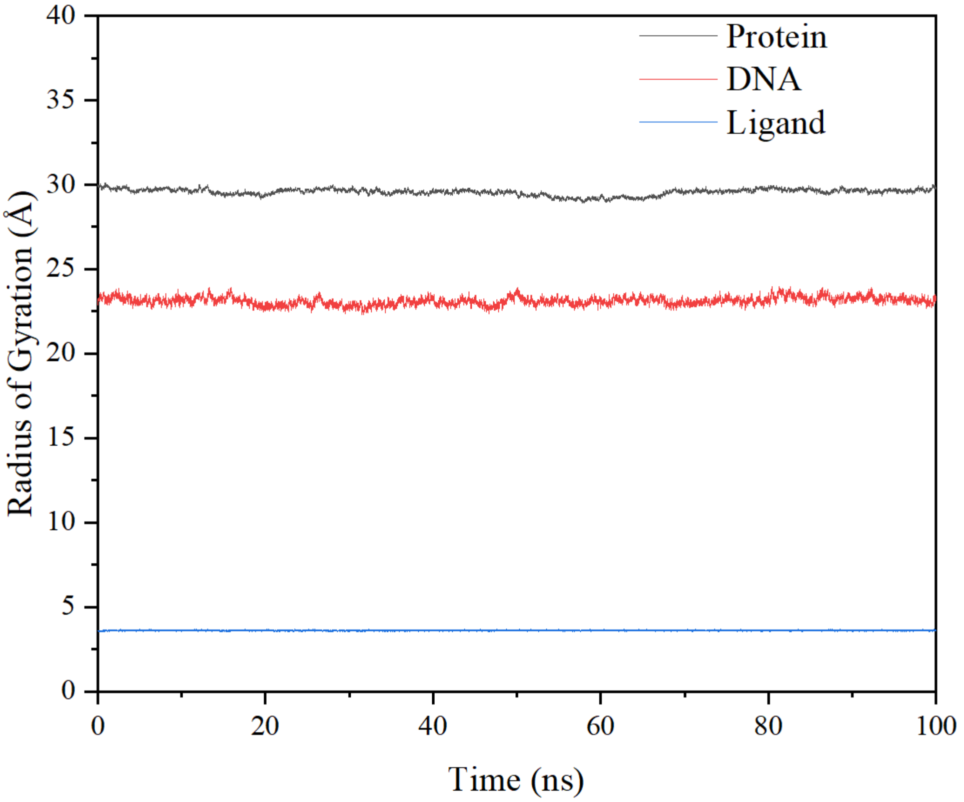

Figure S4. The radius of gyration of protein, DNA, and ligand with 100 ns MD simulations for the TOP1/EVO system.

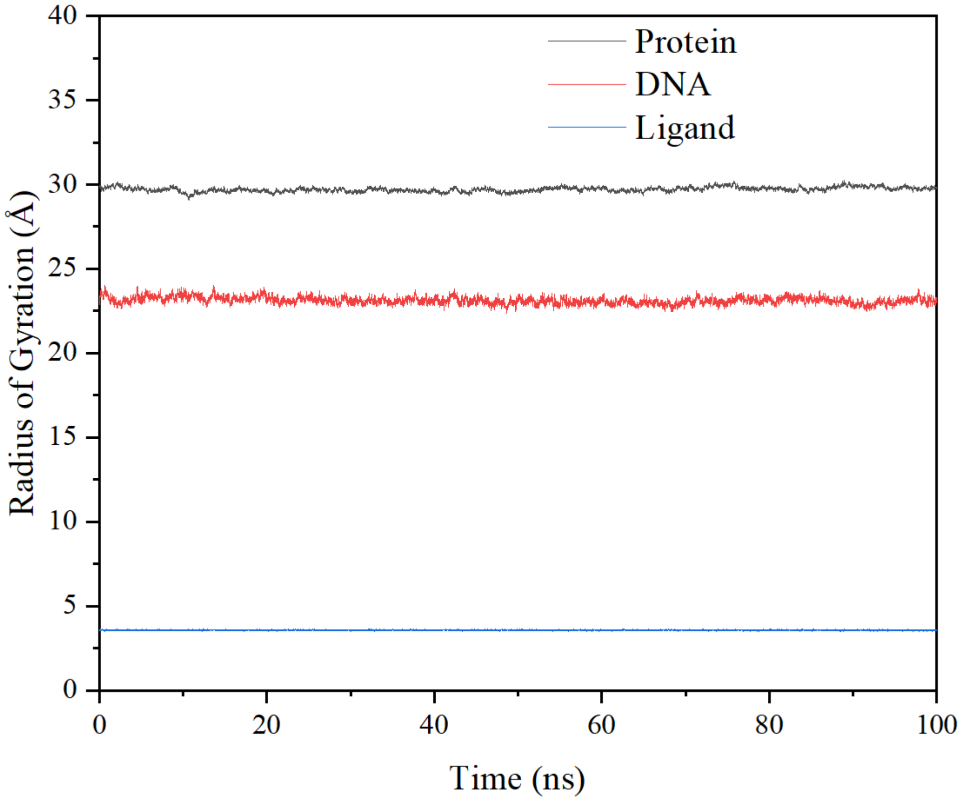

Figure S5. The radius of gyration of protein, DNA, and ligand with 100 ns MD simulations for the TOP1/RUT system.

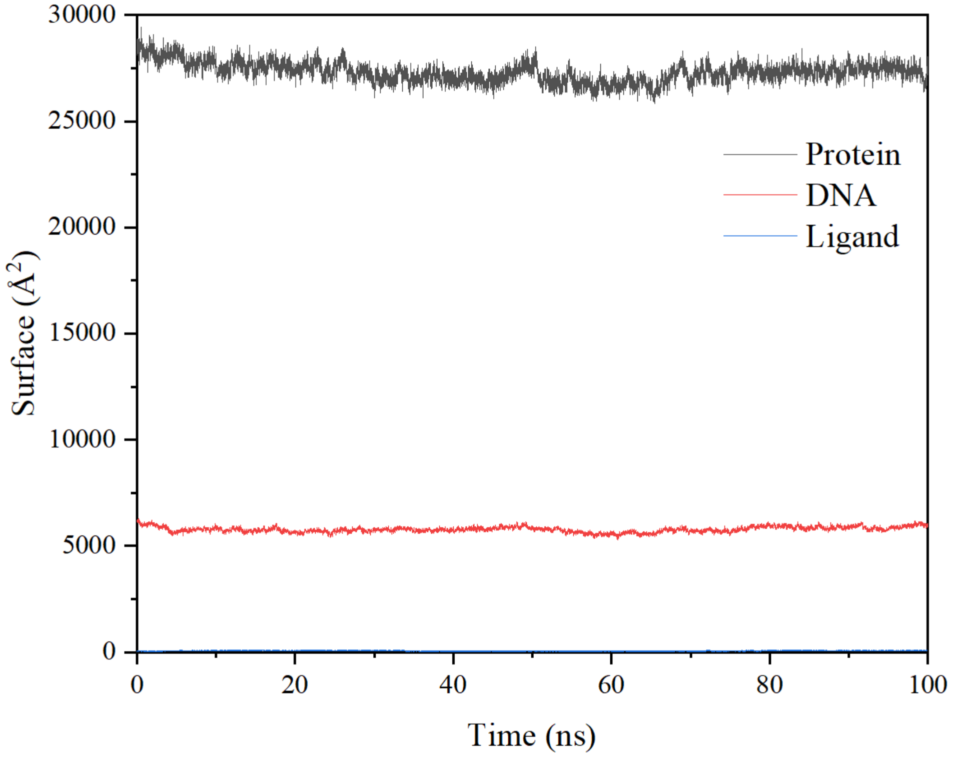

Figure S6. The surface of protein, DNA, and ligand with 100 ns MD simulations for the TOP1/EVO system.

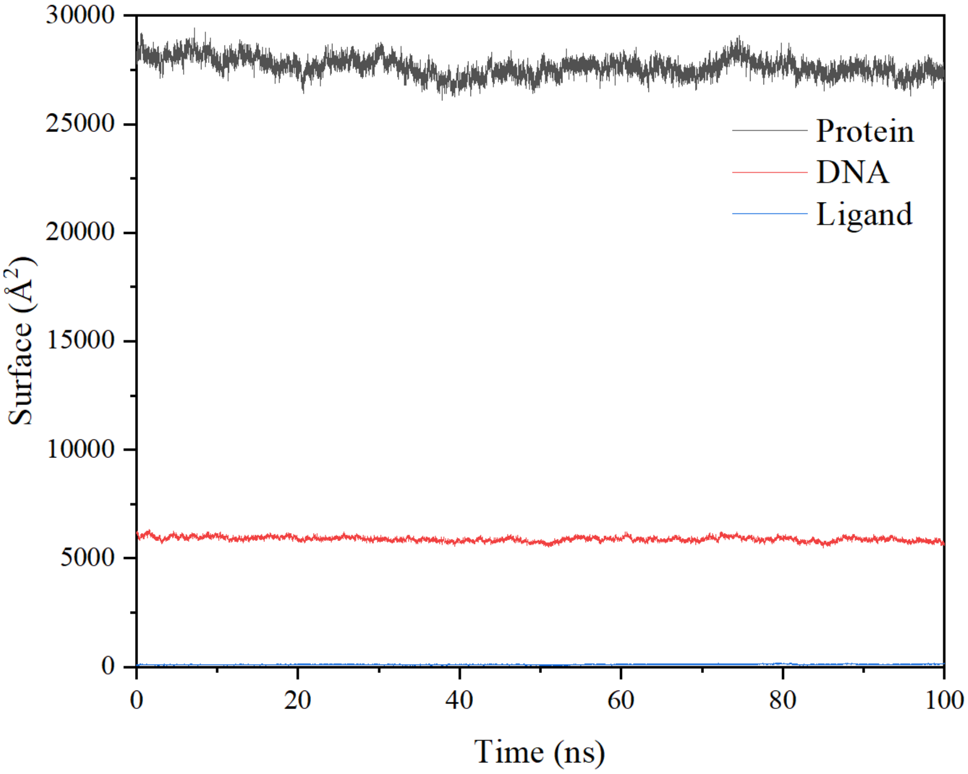

Figure S7. The surface of protein, DNA, and ligand with 100 ns MD simulations for the TOP1/RUT system.

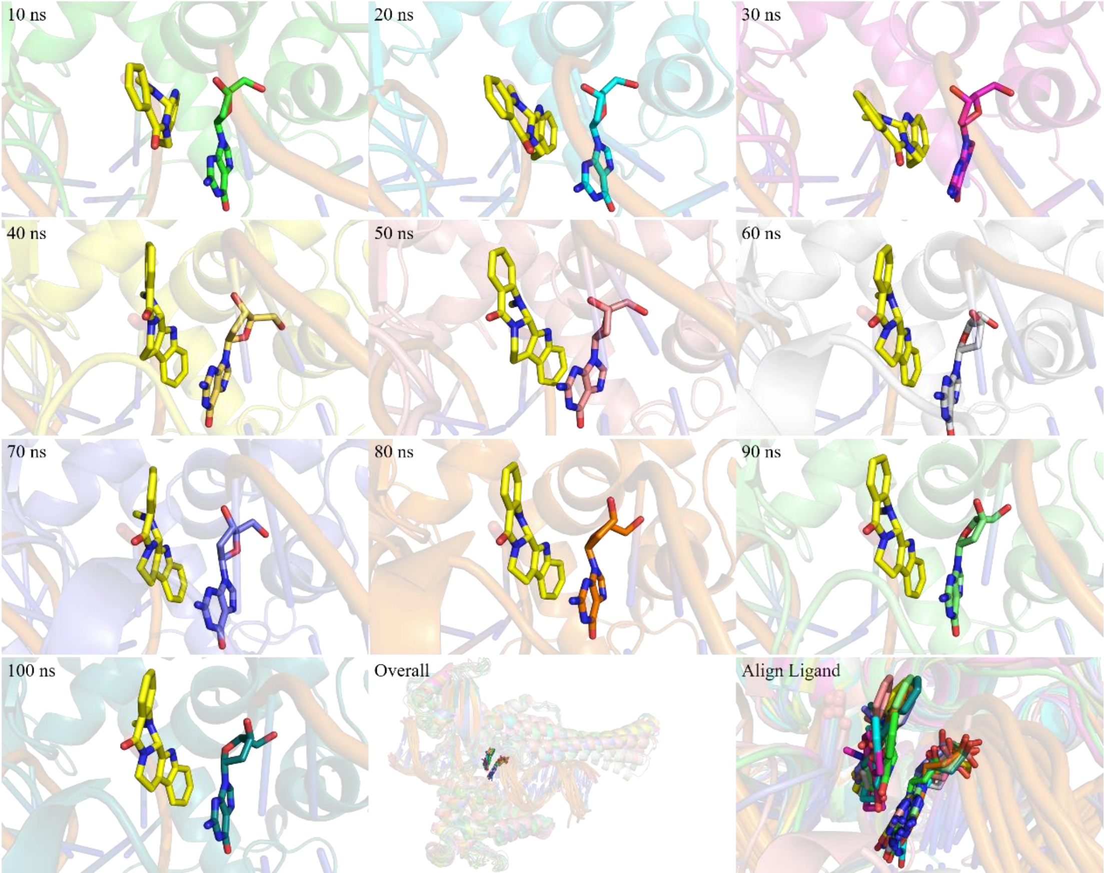

Figure S8. Snapshots of the TOP1/EVO system along the dynamic simulation time for 10, 20, 30, 40, 50, 60, 70, 80, 90, and 100 ns and also their aligned form. For clarity, the water molecules have been removed. The inhibitor is plotted using the stick style (yellow for EVO), while TOP1 is plotted using the cartoon style.

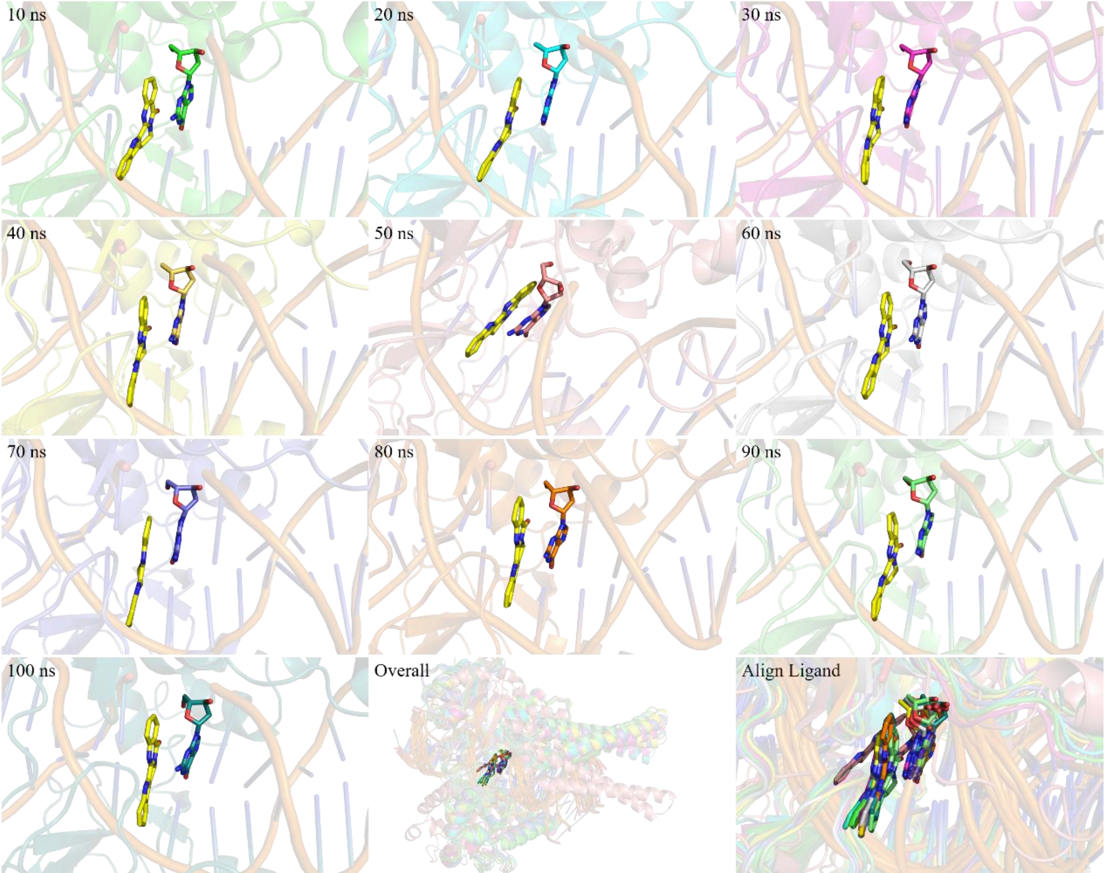

Figure S9. Snapshots of the TOP1/RUT system along the dynamic simulation time for 10, 20, 30, 40, 50, 60, 70, 80, 90, and 100 ns and also their aligned form. For clarity, the water molecules have been removed. The inhibitor is plotted using the stick style (yellow for RUT), while TOP1 is plotted using the cartoon style.

Table S1. Important geometrical parameters of evodiamine (EVO) and rutaecarpine (RUT) obtained by geometry optimization at the B3LYP/6-311++G (d, p) level of theory in gas phase and aqueous media.

	EVO	RUT
Bond distances (Å)		
N6–C5	1.378 (1.367) [1.365]	1.408 (1.399) [1.385]
N6–C7	1.470 (1.472) [1.475]	1.484 (1.488) [1.496]
N6–C13b	1.480 (1.485) [1.472]	1.390 (1.392) [1.394]
N13–C12a	1.382 (1.379) [1.381]	1.376 (1.375) [1.367]
N13–C13a	1.383 (1.382) [1.374]	1.378 (1.380) [1.376]
N14–C13b	1.464 (1.462) [1.443]	1.300 (1.302) [1.296]
N14–C14a	1.427 (1.422) [1.361]	1.380 (1.379) [1.388]
N14–C15	1.476 (1.476) [1.452]	–
C7–C8	1.532 (1.532) [1.535]	1.531 (1.530) [1.509]
C8–C8a	1.498 (1.498) [1.505]	1.497 (1.496) [1.490]
C13a–C13b	1.492 (1.493) [1.515]	1.446 (1.445) [1.443]
Bond angles (deg)		
C5–N6–C7	118.7 (119.4) [117.7]	117.6 (117.9) [117.2]
C5–N6–C13b	116.9 (116.8) [118.2]	121.4 (121.3) [120.6]
C7–N6–C13b	119.9 (120.4) [111.9]	120.4 (120.2) [121.1]
C12a–N13–C13a	108.7 (108.7) [107.6]	108.5 (108.4) [107.9]
C13b–N14–C14a	110.0 (110.4) [118.8]	117.7 (117.9) [117.1]
C13b–N14–C15	115.9 (116.7) [117.8]	–
C14a–N14–C15	113.1 (113.9) [122.3]	–
Torsion angles (deg)		
C13b–N6–C5–C4a	16.5 (15.4) [–22.4]	1.3 (1.6) [2.5]
C13b–N6–C7–C8	–54.2 (–52.2) [–68.3]	–41.4 (–41.2) [–37.6]
C7–N6–C13b–C13a	27.4 (24.3) [59.6]	11.6 (10.9) [13.1]
C5–N6–C13b–N14	–51.8 (–51.3) [44.9]	0.3 (0.1) [0.1]
C14a–N14–C13b–N6	56.4 (56.0) [–41.7]	–0.7 (–0.6) [–0.9]
C13b–N14–C14a–C4a	–29.4 (–28.1) [16.6]	–0.6 (–0.6) [–1.1]
C14a–C4a–C5–N6	11.8 (13.8) [–4.8]	–2.5 (–2.6) [–4.3]
C5–C4a–C14a–N14	–4.6 (–6.8) [8.1]	2.3 (2.3) [3.8]
N6–C7–C8–C8a	48.2 (48.6) [36.9]	46.7 (47.0) [40.7]
C7–C8–C8a–C13a	–23.3 (–24.6) [–6.1]	–27.2 (–27.5) [–24.2]
C8–C8a–C13a–C13b	–1.6 (–1.9) [1.8]	–1.8 (–1.9) [0.2]
C8a–C13a–C13b–N6	1.3 (3.6) [–27.2]	11.2 (11.9) [6.5]

Geometrical parameters obtained in aqueous media are given in parentheses. Experimental bond lengths and bond angles for EVO and RUT obtained from [21] are given in brackets.

Table S2. The traditional and chemical reactivity descriptors (in eV) of evodiamine (EVO) and rutaecarpine (RUT).

Compound	$E_{(HOMO)}$	$E_{(LUMO)}$	ΔE	χ	η	δ	ω	IP	EA
EVO	–5.96	–2.00	3.96	3.98	1.98	0.51	4.00	5.96	2.00
RUT	–5.93	–1.46	4.47	3.69	2.23	0.45	3.05	5.93	1.46

Table S3. Binding free energy of EVO binding with TOP1 protein complexes and decomposition to electrostatic interaction, van der Waals interaction, solvation free energies, and entropy.

Energy (kcal/mol)	Complex		Receptor		Ligand		Delta	
	Average	Std. Dev.*	Average	Std. Dev.	Average	Std. Dev.	Average	Std. Dev.
E_{vdW}	−5631.07	31.47	−5575.61	31.58	−3.77	0.34	−51.68	2.16
E_{ele}	−44314.90	210.87	−44304.17	210.51	−10.49	1.70	−0.24	1.96
E_{GB}	−13084.67	178.59	−13086.93	178.36	−16.67	0.43	18.94	1.70
E_{surf}	239.94	2.01	240.42	2.00	2.51	0.01	−2.99	0.15
G_{gas}	−12654.00	198.27	−12559.15	197.81	−42.94	4.07	−51.92	2.71
G_{solv}	−12844.73	178.67	−12846.52	178.48	−14.17	0.43	15.95	1.69
$E_{gas} + G_{sol}$	−25498.73	76.83	−25405.66	76.68	−57.10	4.08	−35.97	2.32

* The uncertainties for all of terms are included in the parentheses, which were calculated as the root mean square error for all of frames extracted in the MM/GBSA running.

E_{vdW}: contribution to the free energy of binding from van der Waals energy;
E_{ele}: contribution to the free energy of binding from electrostatic energy;
E_{GB}: contribution to the free energy of binding from polar solvation energies;
E_{surf}: contribution to the free energy of binding from nonpolar solvation energies;
G_{gas}: contribution to the free energy of binding from $E_{vdW} + E_{ele}$;
G_{solv}: contribution to the free energy of binding from $E_{GB} + E_{surf}$.

Table S4. Binding free energy of RUT binding with TOP1 protein complexes and decomposition to electrostatic interaction, van der Waals interaction, solvation free energies, and entropy.

Energy (kcal/mol)	Complex		Receptor		Ligand		Delta	
	Average	Std. Dev.*	Average	Std. Dev.	Average	Std. Dev.	Average	Std. Dev.
E_{vdW}	−5674.78	34.58	−5625.92	34.41	−4.43	0.81	−44.43	1.86
E_{ele}	−44389.44	162.43	−44355.42	162.62	−28.32	1.90	−5.70	2.15
E_{GB}	−13014.02	137.39	−13019.41	137.23	−15.76	0.40	21.16	1.93
E_{surf}	239.79	2.77	241.46	2.74	2.69	0.01	−4.36	0.11
G_{gas}	−12579.63	156.44	−12522.65	156.04	−6.85	4.21	−50.14	2.58
G_{solv}	−12774.23	137.35	−12777.95	137.19	−13.08	0.40	16.80	1.92
$E_{gas} + G_{sol}$	−25353.86	76.60	−25300.60	76.12	−19.92	4.23	−33.34	2.13

* The uncertainties for all of terms are included in the parentheses, which were calculated as the root mean square error for all of frames extracted in the MM/GBSA running.

E_{vdW}: contribution to the free energy of binding from van der Waals energy;
E_{ele}: contribution to the free energy of binding from electrostatic energy;
E_{GB}: contribution to the free energy of binding from polar solvation energies;
E_{surf}: contribution to the free energy of binding from nonpolar solvation energies;
G_{gas}: contribution to the free energy of binding from $E_{vdW} + E_{ele}$;
G_{solv}: contribution to the free energy of binding from $E_{GB} + E_{surf}$.

Table S5. Free energy decomposition of the TOP1/EVO complex system at the level of individual residues into contributions from van der Waals energy, electrostatic interaction energy, nonpolar solvation free energy, polar solvation free energy, backbone energy, and side chain energy.

Residue	ΔE_{vdW}	ΔE_{ele}	$\Delta G_{sol,GB}$	$\Delta G_{sol,np}$	$\Delta G_{subtotal}$	$S\Delta G_{subtotal}$	$B\Delta G_{subtotal}$
Protein R364	–1.34	–1.97	2.64	–0.16	–0.81	–0.75	–0.06
Protein K532	–0.46	–1.14	–0.21	–0.02	–1.83	–1.33	–0.50
Protein I535	–1.34	0.03	–0.01	–0.19	–1.51	–1.45	–0.06
Protein H632	–1.25	–0.37	0.98	–0.06	–0.70	–0.55	–0.15
Protein R634	–0.57	0.20	–0.13	–0.09	–0.60	–0.51	–0.09
Protein A715	–0.55	–0.11	0.11	–0.09	–0.65	–0.49	–0.16
Protein T718	–1.01	–3.04	1.18	–0.17	–3.03	–2.77	–0.26
Protein L721	–0.37	0.22	–0.39	–0.03	–0.57	–0.36	–0.21
DNA DT 10	–2.71	–0.70	0.47	–0.23	–3.17	–2.68	–0.49
DNA DT 11	–5.41	–0.84	2.49	–0.46	–4.23	–4.23	0.00

Energies are in kcal/mol.

ΔE_{vdW}: contributions from van der Waals energy;

ΔE_{ele}: contributions from electrostatic interaction energy;

$\Delta G_{sol,GB}$: contributions from polar solvation free energy;

$\Delta G_{sol,np}$: contributions from nonpolar solvation free energy;

$\Delta G_{subtotal}$: contributions from binding free energy;

$S\Delta G_{subtotal}$: contributions from side chain energy;

$B\Delta G_{subtotal}$: contributions from backbone energy.

Table S6. Free energy decomposition for the TOP1/RUT complex system at the level of individual residues into contributions from van der Waals energy, electrostatic interaction energy, nonpolar solvation free energy, polar solvation free energy, backbone energy, and side chain energy.

Residue	ΔE_{vdW}	ΔE_{ele}	$\Delta G_{sol,GB}$	$\Delta G_{sol,np}$	$\Delta G_{subtotal}$	$S\Delta G_{subtotal}$	$B\Delta G_{subtotal}$
Protein R364	–0.96	1.24	–0.77	–0.10	–0.59	–0.54	–0.05
DNA DT 9	–0.35	0.35	–0.45	–0.00	–0.46	–0.32	–0.14
DNA DT 10	–4.53	–0.11	0.71	–0.37	–4.30	–3.74	–0.56
DNA DT 11	–6.52	–0.11	0.65	–0.53	–6.51	–6.51	0.00
DNA DG 12	–0.57	0.43	–0.24	–0.00	–0.38	–0.82	0.44
DNA DC 34	–3.79	–0.03	1.66	–0.31	–2.48	–2.27	–0.21
DNA DA 35	–5.52	–0.91	2.22	–0.37	–4.58	–3.98	–0.60

Energies are in kcal/mol.

ΔE_{vdW}: contributions from van der Waals energy;

ΔE_{ele}: contributions from electrostatic interaction energy;

$\Delta G_{sol,GB}$: contributions from polar solvation free energy;

$\Delta G_{sol,np}$: contributions from nonpolar solvation free energy;

$\Delta G_{subtotal}$: contributions from binding free energy;

$S\Delta G_{subtotal}$: contributions from side chain energy;

$B\Delta G_{subtotal}$: contributions from backbone energy.
